# Effects of near infrared focused laser on the fluorescence of labelled cell membrane

**DOI:** 10.1038/s41598-018-36010-1

**Published:** 2018-12-05

**Authors:** Remy Avila, Elisa Tamariz, Norma Medina-Villalobos, Jordi Andilla, María Marsal, Pablo Loza-Alvarez

**Affiliations:** 1 Centro de Física Aplicada y Tecnología Avanzada, Universidad Nacional Autónoma de México (UNAM), A. P. 1-1010, Juriquilla, 76000 Querétaro Mexico; 2 ICFO-Institut de Ciencies Fotoniques, The Barcelona Institute of Science and Technology, 08860 Castelldefels, Barcelona Spain; 3 Instituto de Ciencias de la Salud, Universidad Veracruzana, Avenicda Luis Castelazo Ayala s/n, Xalapa, 91190 Veracruz Mexico

## Abstract

Near infrared (NIR) laser light can have important reactions on live cells. For example, in a macroscopic scale, it is used therapeutically to reduce inflammation and in a single-cell scale, NIR lasers have been experimentally used to guide neuronal growth. However, little is known about how NIR lasers produce such behaviours on cells. In this paper we report effects of focussing a continuous wave 810-nm wavelength laser on *in vivo* 3T3 cells plasma membrane. Cell membranes were labelled with FM 4-64, a dye that fluoresces when associated to membrane lipids. Confocal microscopy was used to image cell membranes and perform fluorescence recovery after photobleaching (FRAP) experiments. We found that the NIR laser produces an increase of the fluorescence intensity at the location of laser spot. This intensity boost vanishes once the laser is turned off. The mean fluorescence increase, calculated over 75 independent measurements, equals 19%. The experiments reveal that the fluorescence rise is a growing function of the laser power. This dependence is well fitted with a square root function. The FRAP, when the NIR laser is acting on the cell, is twice as large as when the NIR laser is off, and the recovery time is 5 times longer. Based on the experimental evidence and a linear fluorescence model, it is shown that the NIR laser provokes a rise in the number of molecular associations dye-lipid. The results reported here may be a consequence of a combination of induced increments in membrane fluidity and exocytosis.

## Introduction

Plasma membrane dynamics is fundamental in cell secretion, signaling, movement, cell shape changes, cytokinesis, among other cellular processes. Cell membrane is in constant motion due to its characteristic fluid mosaic conformation, where the bilayer of amphiphilic phospholipids diffuse along the membrane plane into specific and heterogeneous membrane domains by translation, rotational motions around the axis perpendicular to the membrane, and by trans-bilayer diffusion in a less extent^1,2^. Cells continually adjust their membrane content and composition by two fundamental processes, the uptake of cell surface membrane called endocytosis, with which fluids or macromolecules may be introduced to the cell, and the fusion of vesicles at the cytoplasmic side of the membrane, called exocytosis, involved in secretion and expression of proteins on the cell membrane^3–5^. Endocytosis and exocytosis are fundamental events in cell protrusion and migration^6–9^.

The interaction of a near infrared (NIR) laser beam with cells has been addressed from many different view angles. From a macroscopic perspective, low levels of NIR laser light are used to reduce pain, inflammation, nerve injuries and to promote tissue regeneration^10–12^. Studies of the influence of low-power helium-neon laser irradiation on selected peripheral blood cells have revealed the importance of photodynamic reactions on the ability of blood to transport oxygen and on immunomodulatory effects on leukocytes^13^. More recently it has been reported that low-intensity NIR laser radiation induces free radical generation and changes in enzymatic and anti-oxidative activities of cellular components^14^. Tightly focused NIR laser radiation has also been used as an attractant of cell projections. This has been described for fibroblasts^15–17^ and neurons^18–25^, opening a new perspective for a possible guidance cue. Although different cellular mechanisms have been suggested to take part in those phenomena, no model has yet gain consensus and in general, the biochemical and biophysical processes that take place when NIR light radiates cells are still poorly understood.

In this paper, we analyze the effect of focused 810-nm laser stimulation on 3T3 fibroblast membrane dynamics. We use FM 4-64, a plasma membrane marker of the family of fluorescent amphiphilic styryl dyes, with a long hydrophobic tail able to interact into the lipid bilayer, whereas the positively charged head group of the molecule prevents the complete insertion into the membrane^26^. Between head and tail, two aromatic rings create the fluorophore. Its quantum yield strongly depends on the solvent polarity, such that in a polar medium like water, the fluorescence is more than two orders of magnitude dimmer than in a non-polar environment, therefore the fluorescence is much higher in the cell membrane than in the media, selectively staining cell surface membrane exposed to the dye^26^. FM dyes have been extensively used to study endo and exocytosis processes in different cell types^26–29^. Here we report previously unknown effects of the NIR laser focussed on the cell membrane: An increase in the fluorescence intensity of the membrane exposed to FM 4-64 at the location of the NIR laser spot, and an increase of the fluorescent recovery after photobleaching (FRAP). Our findings indicate that the fluorescence rise is due to an increase of FM-dye molecules incorporation into the cell membrane, and points towards an increase in membrane dynamic presumably driven by exocytosis events.

## Results

### Observed phenomena

3T3 cells seeded on coverslips were stained with the membrane dye FM4-64. Cells were maintained at 37 °C in a temperature-controlled chamber, immersed in culture medium that contained FM4-64. The complete protocols are described in the Methods Section. Confocal images were obtained by exciting the dye at 561-nm wavelength and detecting fluorescence in the spectral band ranging from 567.5 to 642.5 nm. A continuous wave NIR laser of 810-nm wavelength was optionally focused on a preselected region of interest (ROI) of the cell membrane on the lamella or lamellipodia. The objective used for imaging and focusing the lasers was an oil immersion 60x magnification with numerical aperture of 1.4. The NIR laser power at the exit of the objective was generally 40 mW, except for experiments specifically designed to study the laser power influence.

**Figure 1:**
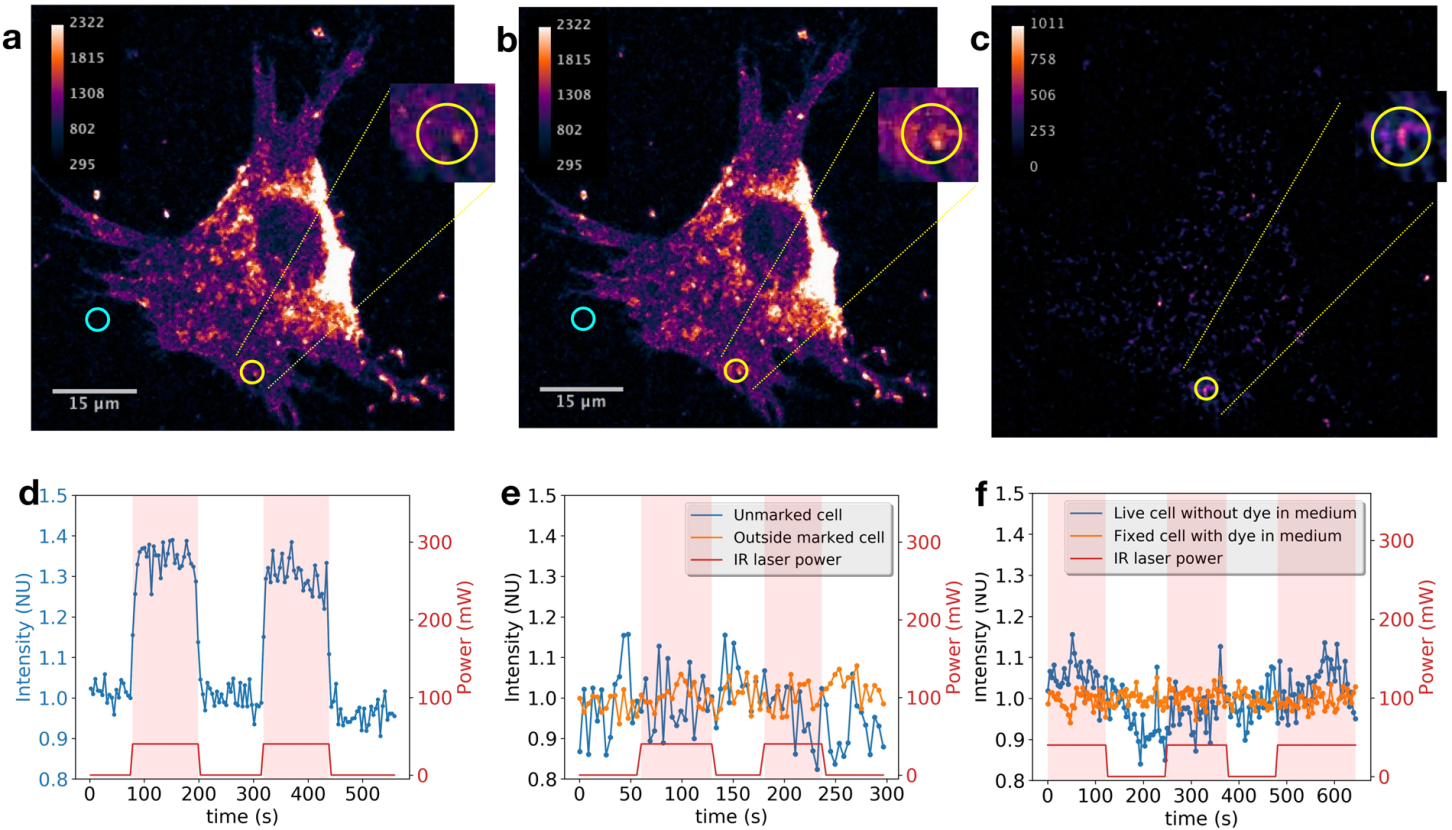
Effect of a 810-nm continuous wave focused laser on fluorescence intensity. (**a**) Confocal image of a 3T3 cell previously stained with FM 4-64, with the presence of dye molecules in the medium. NIR laser is turned off in this image. (**b**) Same as in (**a**) but with the 810-nm laser focused on the centre of the yellow circle which represents the ROI where intensity average is computed. An intensity rise is clearly seen by comparing images in the two magnified regions shown. Cyan circles indicate the regions used to estimate background mean intensity. (**c**) Difference of images b minus a. (**d**) Blue line represents the mean relative intensity (***I***_***rel***_, Equation 1) inside the ROI delimited by the yellow circle. Red line indicates the NIR laser power at the exit of the microscope objective. (**e**) ***I***_***rel***_ measured outside the cell (orange line) and on a cell without staining (blue line). (**f**) Same as in (**c**), but on a sample whose medium is label-free (blue line) and on a fixed cell immersed in medium that contained FM4-46 dye at 1, 1 μM. For the intensity scales in frames (**c**), (**d**) and (**e**) to be comparable among each other, a second normalization is performed on the relative intensity such that plotted values are ***I***_***rel***_(〈***I***_***CELL***_ − ***I***_***BG***_〉/〈***I***_***ROI***_ − ***I***_***BG***_〉) where 〈〉 operator represents a temporal average. See equation (1) for the definition of ***I***_***rel***_.

When the NIR laser is turned on, the fluorescence in the ROI under the NIR radiation influence is seen to increase. An example is shown in Fig. 1a,b. Figure 1c shows the difference of images with laser ON (Fig. 1b) minus the image with laser OFF (Fig. 1a), putting in evidence that within the cell, only in the ROI radiated with the NIR laser the fluorescence increases. Two other examples obtained on different cells are shown in the Supplementary Fig. S1. The ROI on those examples do not contain any bright spot as opposed to the example shown in Fig. 1.

To evaluate this intensity variation compared to the intensity of the entire cell, we define the relative intensity as

1 $${I}_{rel}=\frac{{I}_{ROI}-{I}_{BG}}{{I}_{CELL}-{I}_{BG}},$$

where *I*_*ROI*_, *I*_*CELL*_ and *I*_*BG*_ represent the mean intensities measured within the following three zones: the 4-μm diameter circular ROI where the NIR laser was directed to, the entire cell (manually segmented in each case) and finally a background zone out of the cell. Note that *I*_*rel*_ is independent of the background noise and of the overall fluorescence variation caused for example by photobleaching or fluctuation of the excitation laser power. An example of measured *I*_*rel*_,with and without exposure to the NIR laser, is plotted in Fig. 1d as a function of time. In this figure, one can see that *I*_*rel*_ boosts rapidly when the NIR laser is projected upon the cell and decreases down equally fast to its initial value when the laser is interrupted.

### Control measurements

A series of experiments were performed to discard eventual artefacts. Firstly, we measured *I*_*rel*_ outside the cell. The result is shown by the orange line in Fig. 1e. It indicates two facts: on the one hand, that NIR radiation was not leaking to the detector and on the other hand, that the free dye on the medium does not increase its fluorescence. Moreover, in the experiment shown in the Supplementary Figs S1a and S1b, the laser was located on an edge of the cell, irradiating partly on the cell and partly outside, and the fluorescence increases only on the cell membrane. All those evidences suggest that any fluorescence increase is due to the association dye-membrane, as further explained in the Discussion section. To discard some kind of non-linear autofluorescence of the cell, the NIR laser was directed to the membrane of an unmarked cell. No fluorescence was detected out of the noise. *I*_*rel*_ showed no correlation with the NIR laser intensity (blue line in Fig. 1e).

### Importance of free dye molecules in medium

To further test that the effect under investigation has its origin in some change occurring in the cell-membrane dynamics, we performed measurements on cells that were stained and then fixed with paraformaldehyde at 4%. The dye used for this staining was FM4-64FX, the fixable analogue of FM4-64. During experiments, fixed cells were maintained under similar conditions as live cells, namely, the temperature was kept constant at 37 °C in DMEM plus 10% FBS. The orange line in Fig. 1f is an example of those measurements. It can be seen that the NIR laser does not have any influence on *I*_*rel*_ on fixed cells. This is expected since the membrane of fixed cells is frozen due to covalent cross-links between their components. This strongly suggests the dependence of fluorescence changes in live cells upon molecular motion in the membrane. Moreover, in unfixed and stained cells exposed to NIR laser, but on dye-free medium, no evident increase of fluorescence was observed (Fig. 1f), suggesting that changes in fluorescent intensity mediated by NIR radiation could be due to exocytosis that would increase membrane at the cell surface, and therefore incorporate new dye molecules. Indeed, this scenario is consistent with the fact that if there are no free dye molecules in the medium, no incorporation of new molecules is possible, which could explain the observed absence of fluorescence rise in that case.

### Characterization

**Figure 2:**
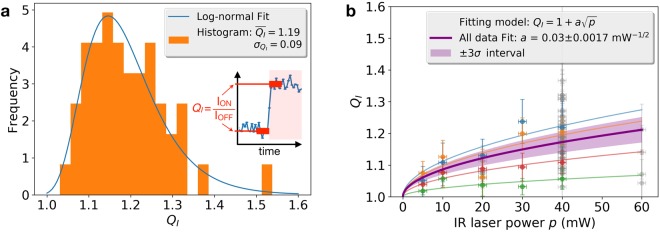
Statistical analysis of the fluorescence rise upon exposure to NIR laser. (**a**) Histogram of the intensity ratio *Q*_*I*_ fitted with a log-normal distribution. The inserted diagram illustrates the definition of *Q*_*I*_ as the ratio of average relative intensities measured when the NIR laser is on and off, the averages being calculated over the 20 s before and after the rise or fall of the NIR laser power. The 48 values of *Q*_*I *_included in the histogram were obtained with an NIR laser power of 40 mW. (**b**) *Q*_*I*_ as a function of laser power, *p*. Blue, orange, red and green data points correspond to individual experiments, each consisting of measuring *Q*_*I*_ as a function of NIR power. Each one of those data sets are fitted with the model indicated in the legend. Grey data points correspond to measurements performed without varying *p*. The complete data set, which consists of 75 values of *Q*_*I*_, is fitted with the purple curve. The width of the light purple zone corresponds to six times the standard deviation of the computed fit.

To quantify and characterise the variation of the relative intensity resulting from the NIR laser influence, we define the ratio

2 $${Q}_{I}=\frac{{\langle {I}_{rel}\rangle }_{{T}_{ON}}}{{\langle {I}_{rel}\rangle }_{{T}_{OFF}}},$$

where the average in the denominator and numerator is performed over periods *T*_*OFF*_ and *T*_*ON*_ that correspond to the 20-s period closer to the change of state of the NIR laser between OFF and ON, respectively. This construction is sketched in the inset of Fig. 2a. In that same figure we show the histogram of the experimental values of *Q*_*I*_, which is fitted with a log-normal probability (see Methods). The expected value and the variance of a random variable that has the fitted probability density function are *E*(*Q*_*I*_) = 1.19 and *V*(*Q*_*I*_) = 0.0092, respectively, which correspond fairly well with the mean and the variance of the data $\overline{{Q}_{I}}=1.19$ and ${\sigma }_{{Q}_{I}}^{2}=0.0081$. This means that the expected mean increase of the relative fluorescence intensity is 19%, with a standard deviation of $\sqrt{0.0092}\times 100=9.6 \%$.

One evident question that comes to mind is how the fluorescence rise depends on the power of the NIR laser. We measured *Q*_*I*_ for NIR powers ranging from 5 to 40 mW, in four independent experiments. Blue, orange, red and green data points plotted in Fig. 2b correspond to those individual experiments. Each data set is fitted with a function of the form

3 $${Q}_{I}(p)=1+a\sqrt{p},$$

*p* being the NIR laser power and *a* the free parameter for the fit. The choice of a square root dependence responds to a suspected relation between laser power and thermal velocity of the molecules (see Discussion section). All four experiments follow reasonably well this functional dependence, each with a different value of *a*. In Fig. 2b, the grey data points represent the measured values of *Q*_*I*_ that were obtained in experiments where *p* was kept constant either at 40 or 60 mW. All the data put together is fitted with same equation (3), obtaining *a* = 0.03 ± 0.0017mW^−1/2^. The fit is plotted with the purple thick line in Fig. 2b.

### Fluorescence recovery after photobleaching

**Figure 3:**
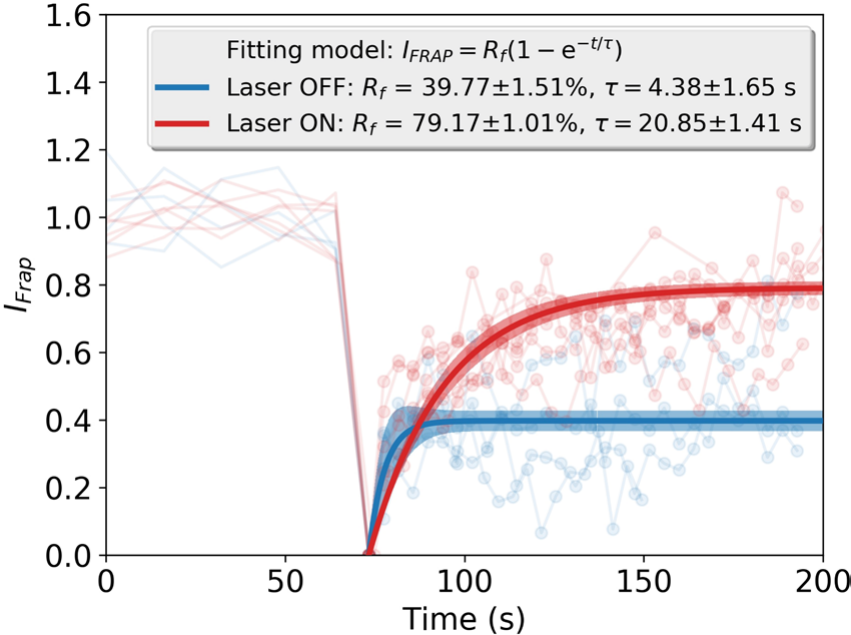
Fluorescence Recovery After Photobleaching (FRAP). Recovered Intensity *I*_*FRAP*_ (see Methods section) is plotted as a function of time for seven experiments with NIR laser (red) and four experiments without NIR laser (blue). When turned on, the NIR laser was focused on the ROI of the cell membrane where the FRAP experiment was carried on. Photobleaching occurs when *I*_*FRAP*_ = 0. After that moment, fluorescence in the bleached ROI begins to recover. Bold curves are fits to the data following the standard model indicated in the legend, where *R*_*f*_ and *τ* account for the fraction of intensity recovery and half recovery time, respectively. The width of the light blue and red zones for each instant correspond to the standard deviation of the computed fit.

In order to obtain more data about the kinetics of fluorescent changes mediated by NIR laser, the recovery of the fluorescence after photobleaching was measured in 11 experiments, in 7 of which the NIR laser was focused on the bleached region during the entire experiment, and in 4 of them the NIR laser was blocked, also during the whole experiment. The measured intensity was doubly normalized (see Methods) so that the resulting intensity, *I*_*FRAP*_, equals unity before photobleaching and is corrected for photofading caused by imaging after the bleach^30^. The results are shown in Fig. 3, where the red and blue plots correspond to experiments done with and without the NIR laser. All the data in each of those two categories were fitted to an exponential function as indicated in the legend of Fig. 3 and in the Methods section. Fluorescence recovery when the NIR laser is acting on the cell is almost twice as large as when the NIR laser is off, as deduced by the corresponding fitted values of *R*_*f*_: 79 ± 1 and 40 ± 1.5. The half recovery time given by the fit is much larger when the NIR laser is on (*τ* = 20 ± 1.4 s) than when it is off (*τ* = 4 ± 1.6 s). Those results indicate that a higher proportion of FM molecules are incorporated at the bleached area when the NIR laser is ON, although with a smaller rate than when laser is OFF.

## Discussion

**Figure 4:**
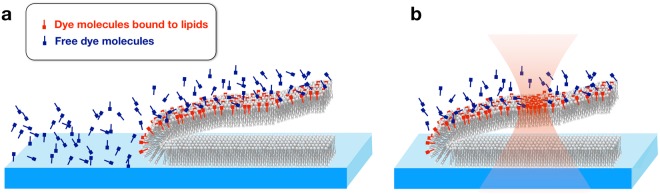
Plasma membrane stained with the fluorescent dye FM4-64. Dye molecules that are adhered to the lipids of the membrane are represented in red and those that move freely in the culture medium are drawn in blue. The NIR respective quantum yields are Φ_*a*_ and Φ_*b*_. It is well known that FM4-64 fluoresces significantly more when associated to lipids than on polar solvent like water (Φ_*a*_ ≫ Φ_*b*_). (**a)** Out of the cell, only molecules with quantum yield Φ_*b*_ are found, whereas in the vicinity of the cell membrane both types of molecules coexist. (**b**) A NIR laser beam focused on the membrane provokes an increase of the proportion of dye molecules that are bound to lipids and the consequent rise of the fluorescent intensity when the cell is imaged in confocal microscopy.

FM dyes are known to be brighter when associated with lipids (or other non-polar molecules such as prolactin proteins^31^) than in aqueous solution. The quantum yield Φ_*a*_ of [dye-membrane] is considerably larger than that of the dye alone, Φ_*b*_. A schematic representation of the distribution of the FM4-64 molecules is given in Fig. 4a. Assuming that the fluorescence measurements are performed in the linear regime so that photobleaching (except of course FRAP experiments) and quenching effects can be neglected, the measured fluorescence intensity can be modelled as:

4 $$I=\eta \mathop{\int }\limits_{{\rm{V}}}{\rm{d}}{\bf{r}}\,{n}_{V}({\bf{r}})[{\rm{\alpha }}({\bf{r}}){{\rm{\Phi }}}_{a}+(1-{\rm{\alpha }}({\bf{r}})){{\rm{\Phi }}}_{b}]+{I}_{dark}.$$

In this expression, all the photons emitted within the volume V are added up. *n*_*V*_ represents the volumetric density of FM4-64 dye molecules, which is a function of the three-dimensional position vector **r**. the number of dye molecules that are bound to lipids is represented by α(**r**). The factor *η* depends linearly on the excitation laser power, the voltage of the photomultiplier detector and of the dwell time. *I*_*dark*_ represents the intensity measured in dark conditions (absence of signal). Using this model and our experimental evidence about the NIR laser impact on fluorescence, we show (Methods) that the NIR radiation is not modifying the properties of dye and that the fluorescence variation due to the NIR laser originates in an increase of the number of fluorescent associations [dye-lipid] in the ROI (See Fig. 4b). This could occur presumably by a combination of a number of effects like membrane fluidity alteration, a modification in exo- and endocytosis rates, and exchange of dye molecules between the lipid bilayer and the medium.

Most interestingly, we show (Methods) that

5 $${Q}_{I}\equiv \frac{{I}_{re{l}_{ON}}}{{I}_{re{l}_{OFF}}}=\frac{{\alpha }_{RO{I}_{ON}}}{{\alpha }_{RO{I}_{OFF}}}.$$

That is, *Q*_*I*_ equals the ratio of the fraction of dye molecules bound to lipids when the NIR is ON (${\alpha }_{RO{I}_{ON}}$) over that when the NIR is OFF (${\alpha }_{RO{I}_{OFF}}$), which gives a direct mean of quantifying the increment of dye molecules that are bound to lipids in the membrane as a consequence of the NIR radiation. The statistical analysis of *Q*_*I*_ (Fig. 2a) indicates that the NIR laser at 40 mW induces a mean increment of bounds [dye-lipid] of 19%.

**Figure 5:**
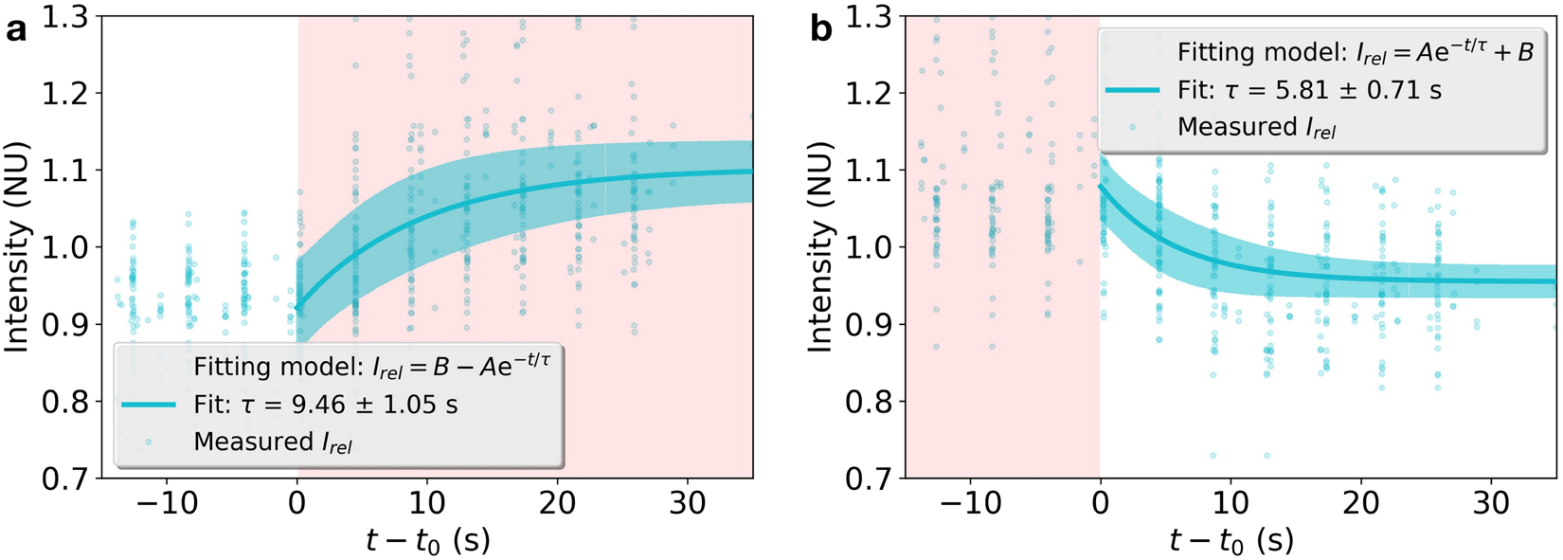
Timescale of fluorescence increase (**a**) and decay (**b**) as a result of NIR laser irradiation. Measured relative intensity values are represented by light cyan dots. The horizontal axis represents time minus time *t*_0_ at which the laser is turned on (**a**) and off (**b**). For positive values of *t* − *t*_0_, an exponential model is fitted to the data as indicated in the respective legend, where the time constant of each exponential model is also indicated. The width of the cyan zones for each instant correspond to six times the standard deviation of the computed fit.

This scenario is further supported by the much higher FRAP when the NIR laser is present than when it is not. When the NIR laser is off, the fluorescence recovery can be understood in the classical way where fluorescent molecules within the membrane diffuse into the bleached spot and fluorescence recovers up to a certain level from which the mobile fraction of fluorescent molecules is determined^30,32^. When the NIR laser is acting on the membrane, the fluorescence recovery could come from two sources: in one hand, membrane-bound dye molecules diffusing in two dimensions and in the other hand, free dye molecules diffusing in three dimensions in the medium and getting bound to extra membrane available probably by exocytosis, increasing the fluorescent amount, as reported previously^29^. To investigate whether or not the timescales of the fluorescence intensity decay and rise are compatible with the possible explanations we are advancing, we gathered all the rise measurements in one plot and all the decay measurements in another plot and fitted exponential functions. The results are shown in Fig. 5. The fluorescence rise and decay are well fitted by exponential functions with a time constants equal to 9.46 ± 1.05 s and 5.81 ± 0.71 s, respectively. Those values are consistent with endo and exocytosis events that can take place in timescales shorter than tens of seconds^29,33^ and even milliseconds^34^, and that can even happen simultaneously. The measured timescale of the fluorescence decay is longer than the timescales that have been reported for FM dye dissociation from lipids that range from milliseconds to seconds, depending on the dye concentration and membrane temperature^35^.

It has been shown^36^ that 810-nm laser radiation, with power ranging from 10 to 400 mW, can induce an increase in the fluidity of isolated erythrocytes membranes and activate membrane ATPasa, in a laser-power-dependent way. In our results, NIR irradiation could similarly be increasing membrane fluidity, however, unlike experiments on isolated cell membrane, our *in vivo* protocol allowed us to observe an extra contribution to fluorescent recovery that could be explained by other cell membrane events as exocytosis. Interestingly, it has been reported^37–39^ that low-energy NIR laser radiation induces the secretion and release of interleukins and growth factors in several cells like macrophages, keratinocytes and fibroblasts. The same kind of radiation can have a therapeutic effect in inflammation and wound healing by secretion of inflammatory mediators^40–43^. However, to our knowledge, no direct experimental evidence of cellular mechanisms driving secretion is available.

Also, we find that *Q*_*I*_ (or, from Equation 5, the fraction of dye molecules adhered to membrane lipids) can be adjusted with a square root dependence of the laser power (Fig. 2b). This functional relation is reminiscent of the speed of molecules in Brownian motion as a function on the thermal energy in a fluid. An increase of temperature due to the NIR laser would be consistent with the increase of dye molecules being introduced between lipid molecules into the membrane. However, several authors^44–46^ have reported that NIR optical tweezers, which use a similar laser beam as that used here, provoke a temperature increase of only a few degrees Kelvin on biological samples immersed in water, where the typical temperature ranges from 298 to 310 °K. For example, a steady temperature increase of 1.9 °C is calculated^46^ for a 1064-nm 100 mW laser in the focus of a microscope objective of 1.3 numerical aperture, in water, after only a few milliseconds. Considering the at that wavelength the absorption coefficient of water is approximately 5 times higher than at 810-nm^47^, that the spot area given by a 1.2 N.A. objective is 1.36 times larger than that given by a 1.4 N.A. (as ours), and that the laser power used in our experiments is 40% of that used by Catalá *et al*.^46^, it is safe to consider that the temperature increase due to the 810-nm laser is lower than 1 °C. Such a small increase in temperature could hardly account for the measured increase in dye-lipid bounds, under the light of, for example, the results of a study of the temperature dependence of the structure of Dioleoylphosphatidylcholine bilayers^48^. It has been reported^49^ that a large focalized temperature increase in a mono-layer lipid membrane makes the membrane permeable to ions even in the absence of ion channels. In that investigation, the authors illuminate, with a visible laser, gold nanoparticles that upon excitation of the plasmon resonance transfer heat to the membrane. In our case, either the local temperature rise is much larger than the few Kelvins expected from the literature, or there are additional phenomena involved.

## Conclusion

Our study shows that a near NIR laser focused on a live 3T3 cell promotes binding of free dye molecules floating in the culture medium with lipid molecules in the membrane. Such a result is consistent with the laser modifying the molecular dynamics in the membrane presumably due to two factors: membrane fluidity and exocytosis, however definitive explanations need further investigations. To the best of our knowledge, this is the first time that the influence of a focused NIR laser on the lipid dynamics of a live cell plasma membrane is demonstrated. A complete understanding of NIR laser radiation on living cells is far from being achieved. Studies of the rheological properties of bilayer lipid membranes under the influence of an NIR laser can bring important clues. Those biophysical experiments should be complemented with investigations of the effect of NIR laser radiation on the biochemistry of molecular receptors and signalling processes for wider perspective to be attained. Understanding the interaction of NIR laser with live cells can lead to the development of innovative protocols and therapies.

## Methods

### Reagents

FM 4-64 Dye (*N*-(3-Triethylammoniumpropyl)-4-(6-(4-(Diethylamino) Phenyl) Hexatrienyl) Pyridinium Dibromide) and its fixable analog FM 4-64 FX were purchased from Invitrogen. Hank’s Balanced Salt Solution (HBSS), Dulbecco’s modified Eagle’s medium (DMEM), bovine-inactivate fetal serum (FBS), N-2-hydroxyethylpiperazine-N-2-ethane sulfonic acid (HEPES 1 M), Trypsin-EDTA 1x and Penicillin/streptomycin were purchased from Gibco BRL Life Technologies. Collagen Type I, phosphate-buffered saline (PBS), Paraformaldehyde (PFA) and Dimethyl Sulfoxide (DMSO) were purchased from Sigma-Aldrich.

### Cell culture

NIH3T3 cells were cultured in DMEM supplemented with 10% FBS and 1% penicillin/streptomycin in a 37 °C and 5% CO_2_ incubator. The cells were gently washed with PBS 1x and detached from the culture dish by exposing them to 1 mL Trypsin-EDTA 1x for 5 min at 37 °C with 5% CO_2_. Then 3 mL DMEM was added to the culture and the solution obtained was centrifuged at 1000 rpm for 5 min. The pellet was drained and then homogenized with 1 mL supplemented DMEM. Cell density was measured using a hemocytometer after staining with trypan blue. A stock of 2 × 10^4^ cells was maintained in supplemented DMEM medium and 3 × 10^3^ cells were cultured on coverslips previously treated with 2 µg/mL of collagen type I kept at 37 °C in a humidified atmosphere with 5% CO_2_ for 2 days. The treatment of sterile coverslips with Collagen consisted in adding 200 µL of the solution on the coverslip and incubated at 37 °C for 1 h and washed once with PBS.

### Sample preparation

Cells were labelled by incubating them for 20 minutes at 37 °C in a solution of FM4-64 at a final concentration of consisting of 5 µg/mL in HBSS (FM 4-64 staining solution). After the staining incubation, the coverslip was installed inside a Chamlide magnetic chamber filled with 1.5 mL of a solution constitued of DMEM plus 10% FBS, buffered by HEPES 10 mM plus 1 µL of FM 4-64 solution at 1 mg/mL in DMSO (FM 4-64 register solution). The chamber was placed in a Tokai hit top stage incubator to maintain the sample at *37 °C*. For the control experiments where the cell medium was free of dye molecules, prior to putting the coverslip inside the magnetic chamber, the cells were gently washed with PBS 1x and the chamber was filled with DMEM plus 10% FBS, buffered by HEPES 10 mM. For the experiments on fixed cells, the cells were first labelled following the same procedure as described above but instead of using FM 4-64 we used FM 4-64 FX. Then cells were fixed with PFA at 4%.

### Optical Setup

**Figure 6:**
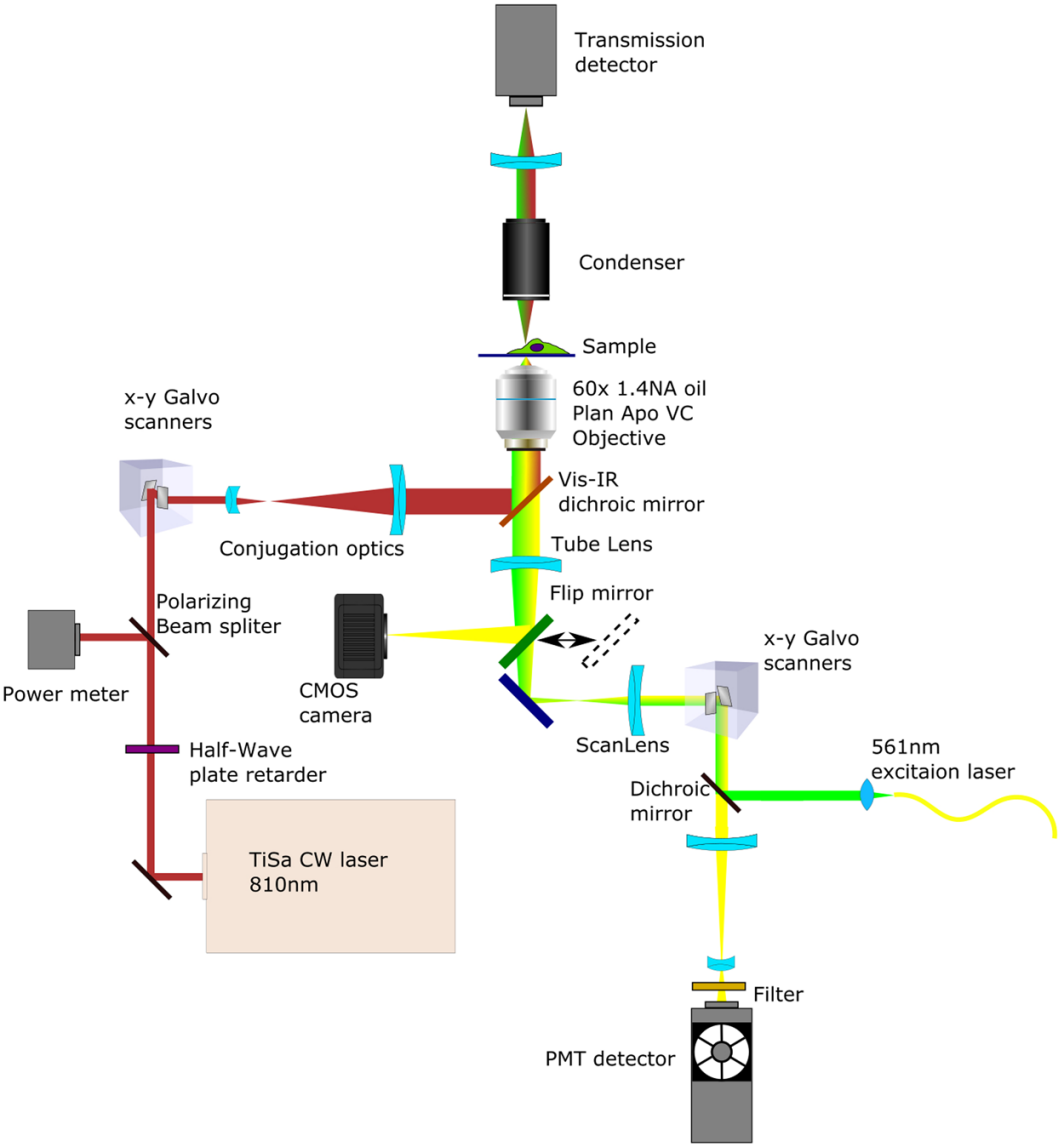
Optical layout of the experiments. The instrument is based on a Nikon Eclipse Ti confocal microscope. A Ti:Sa laser operating in CW mode provides the 810-nm radiation that enters through one of the rear ports of the microscope and is reflected by a dichroic mirror. The infrared spot position is controlled with galvanometric mirrors labelled Galvo X and Galvo Y. Lenses 1 through 4 ensure beam expansion and conjugation of the galvanometric mirrors to the objective pupil. The 810-nm laser power is controlled with a half-wave plate and a polarising beam splitter that sends one polarization direction to a power meter and the other to the microscope. The sample is mounted inside a chamber with temperature held at 37 °C using a Tokai Hit stage top incubator.

A scheme of the optical layout is provided in Fig. 6. The instrument is based on a Nikon Eclipse Ti inverted microscope equipped with a C1si confocal system. The CW Ti:Sa laser is positioned using a galvanometric mirrors pair entering through the rear port. The NIR beam is imaged in the back focal plane of the objective by means of the conjugation optics and reflected by the short-pass (FF720-SDi01-25 × 36) dichroic mirror. The power of the NIR laser is controlled by measuring the power reflected by the power meter after a polarizing BS cube when rotating a half-wave retarder. Transmission images can be acquired either using the transmission detector when using the confocal unit or using a CMOS camera (DCC1545M-Thorlabs) when illuminating with the standard halogen lamp. During the experiments the sample is mounted inside a chamber with temperature held at 37 °C using a Tokai Hit stage top incubator.

### Measurements

#### Fluorescence Intensity measurements

The microscope control and image acquisition were done using software NIS-Elements (Nikon Instruments Microscopes and Digital Imaging Systems) software. Fluorophores were excited with the 561-nm laser of the confocal microscope at 5% of its maximum power. Images consisting of 512 × 512 pixels were acquired every 4.52 s with unidirectional scan. For FRAP experiments, five frames every 15.96 s were taken prior to photobleaching, then the ROI on the sample was expose three times to the excitation laser at its maximum power. After that, images for the measurements of the fluorescence recovery were obtained as described before (every 4.52 s), for at least two more minutes. The detector of transmitted light was used as a monitor of the NIR laser status (ON or OFF).

#### NIR laser power measurements

The polarizing beam splitter (Fig. 6) in the 810-nm laser path divides the laser radiation in two complementary parts, the proportion of which is determined by the rotation angle of the half-wave plate retarder. The power that is directed to the microscope equals the total laser power minus the power that is directed to the power-meter. To take into account losses in the optical path, prior to the experiments, the power at the power-meter was cross-calibrated to the power measured at the exit of the microscope objective with another power-meter. This calibration was then used to determine the NIR laser power at the sample by measuring the power at the power-meter shown in Fig. 6.

### Data analysis and fitting

#### Log-normal probability density function

The histogram of *Q*_*I*_ values (Fig. 2a) is fitted with a log-normal probability function of the form:

6 $$f({Q}_{I})=\frac{1}{({Q}_{{\rm{I}}}-{Q}_{{\rm{c}}}){\sigma }_{I}\sqrt{2\pi }}{{\rm{e}}}^{\frac{-{(\mathrm{log}({Q}_{I}-{Q}_{c})-{\mu }_{I})}^{2}}{2{{\sigma }_{I}}^{2}}}$$

The parameter values that produced the best fit were *Q*_*c*_ = 0.92, *μ*_*I*_ = −1.37 and *σ*_*I*_ = 0.34. Fitting was computed using SciPy statistical lognorm package under python. Following the documentation of that package, the expected value *E*(*Q*_*I*_) and the variance *V*(*Q*_*I*_) of such a random variable are calculated by

7 $$E({Q}_{I})={{\rm{e}}}^{{\mu }_{I}+\frac{{{\sigma }_{I}}^{2}}{2}}+{Q}_{c}$$

8 $$V({Q}_{I})=({{\rm{e}}}^{{{\sigma }_{I}}^{2}}-1){{\rm{e}}}^{2{\mu }_{I}+{{\sigma }_{I}}^{2}}.$$

For the parameter values given by the fit, we obtain *E*(*Q*_*I*_) = 1.19 and *V*(*Q*_*I*_) = 0.0092.

#### FRAP

The analysis of the FRAP was based on the double normalization of intensity measurements, which involves normalization of the recovery signal to the average prebleach signal and, at the same time, takes into account the loss of signal due to photofading during postbleach imaging. We also correct for the background intensity. Let us define the background corrected mean intensities in the ROI and in the whole cell as:

9 $${I}_{ROI}^{\text{'}}(t)={I}_{ROI}(t)-{I}_{BG}(t)$$

10 $${I}_{CELL}^{\text{'}}(t)={I}_{CELL}(t)-{I}_{BG}(t)$$

The ROI is photobleached at time *t*_*p*_. The normalized intensity is:

11 $${I}_{FRAP}(t)=\frac{{I}_{ROI}^{\text{'}}(t)-{I}_{ROI}^{\text{'}}({t}_{p})}{{\langle {I}_{ROI}^{\text{'}}\rangle }_{t < {t}_{p}}-{I}_{ROI}^{\text{'}}({t}_{p})}\times \frac{{\langle {I}_{CELL}^{\text{'}}\rangle }_{t < {t}_{p}}\,}{{I}_{CELL}^{\text{'}}(t)}$$

where the symbol 〈〉$t < {t}_{p}$ represents averaging before photobleaching. *I*_*FRAP*_(*t*) is fitted to *R*_*f*_(1 − *e*^−*t*/*τ*^).

### Demonstration of insensitivity of quantum yields **Φ**_***a***_ and **Φ**_***b***_ to the NIR laser

A good approximation to Equation 4 is obtained by considering volume V being a cylinder of base A and altitude h. The fluorescence intensity averaged over area A can be written as:

12 $${I}_{A}\equiv \frac{I}{A}=h\eta [{{\rm{\Phi }}}_{a}\bar{\alpha {n}_{A}}+{{\rm{\Phi }}}_{b}\bar{(1-\alpha ){n}_{A}}]+\bar{{I}_{dark}}$$

The bar indicates an average over surface A. Considering that the surface density of dye molecules *n*_*A*_ and α are statistically independent, $\overline{\alpha {n}_{A}}=\bar{\alpha }\,\overline{{n}_{A}}$ and $\overline{(1-\alpha ){n}_{A}}=(1-\bar{\alpha })\overline{{n}_{A}}$. Moreover, to simplify mathematical notations, let the averaging over a region be noted by a subindex such that *α*_*A*_ ≡ ${\alpha }_{A}\equiv \bar{\alpha }$ . We can safely consider that the spatial distribution of the dye molecules is homogeneous and simply designated by $n\equiv \overline{{n}_{A}}$. The same consideration applies to the dark current so that ${I}_{dark}=\bar{{I}_{dark}}$ for any region. Equation 12 is thus simplified as:

13 $${I}_{A}=h\eta [{{\rm{\Phi }}}_{a}{\alpha }_{A}n+{{\rm{\Phi }}}_{b}(1-{\alpha }_{A})n]+{I}_{dark}.$$

#### Measurements out of the cell

For the control experiments performed out of the cell (see Fig. 1e), the relative intensity used is:

14 $${I}_{re{l}_{OUT}}=\frac{{I}_{OUT}}{{I}_{BG}}$$

The numerator is obtained from Equation 13 by replacing subindex *A* by *OUT*. Since out of the cell the abundance of lipids can be neglected, ${\alpha }_{OUT}\simeq 0$, thus

15 $${I}_{OUT}\simeq h\eta n{{\rm{\Phi }}}_{b}+{I}_{dark}.$$

The only parameter in last equation that could imaginably vary as a consequence of the NIR laser is the quantum yield of dye molecules unbound to lipids Φ_*b*_ which would take values Φ_*bON*_ or Φ_*bOFF*_ depending on the NIR laser status. The intensity on the background is always

16 $${I}_{BG}\simeq h\eta n{{\rm{\Phi }}}_{{b}_{OFF}}+{I}_{dark}.$$

As ${I}_{re{l}_{OUT}}$ is not seen to depend on the NIR laser power, we conclude that this radiation does not affect the quantum yield of the dye molecules that are unbound to lipids:

17 $${{\rm{\Phi }}}_{{b}_{ON}}={{\rm{\Phi }}}_{{b}_{OFF}}={{\rm{\Phi }}}_{b}.$$

#### Measurements on the cell

The relative intensity measured on the cell membrane with NIR laser ON and OFF is respectively given by

18 $${I}_{re{l}_{ON}}=\frac{{I}_{RO{I}_{ON}}-{I}_{BG}}{{I}_{CEL{L}_{ON}}-{I}_{BG}}$$

19 $${\rm{and}}\,{I}_{re{l}_{OFF}}=\frac{{I}_{RO{I}_{OFF}}-{I}_{BG}}{{I}_{CEL{L}_{OFF}}-{I}_{BG}}.$$

Considering that the cell area is much larger than the area influenced by the NIR spot, it is reasonable to make the approximation ${I}_{CEL{L}_{ON}}\simeq {I}_{CEL{L}_{OFF}}={I}_{CELL}$. The three terms involved in Equation 18:

20 $${I}_{RO{I}_{ON}}=h\eta [{{\rm{\Phi }}}_{{a}_{ON}}{\alpha }_{RO{I}_{ON}}n+{{\rm{\Phi }}}_{b}(1-{\alpha }_{RO{I}_{ON}})n]+{I}_{dark}$$

21 $${I}_{CELL}=h\eta [{{\rm{\Phi }}}_{a}{\alpha }_{CELL}n+{{\rm{\Phi }}}_{b}(1-{\alpha }_{CELL})n]+{I}_{dark}$$

22 $${I}_{BG}\simeq h\eta n{{\rm{\Phi }}}_{b}+{I}_{dark}$$

lead to a simplified expression for the relative intensity for the NIR laser ON:

23 $${I}_{re{l}_{ON}}=\frac{({{\rm{\Phi }}}_{{a}_{ON}}-{{\rm{\Phi }}}_{b}){\alpha }_{RO{I}_{ON}}}{({{\rm{\Phi }}}_{a}-{{\rm{\Phi }}}_{b}){\alpha }_{CELL}}.$$

Equivalently, the relative intensity for the case where the NIR laser is OFF becomes

24 $${I}_{re{l}_{OFF}}=\frac{({{\rm{\Phi }}}_{a}-{{\rm{\Phi }}}_{b}){\alpha }_{RO{I}_{OFF}}}{({{\rm{\Phi }}}_{a}-{{\rm{\Phi }}}_{b}){\alpha }_{CELL}}=\frac{{\alpha }_{RO{I}_{OFF}}}{{\alpha }_{CELL}}.$$

Now, we find that when there is no free dye in the medium, the NIR laser does not induce any change in the relative intensity (see Fig. 1f), thus ${I}_{re{l}_{ON}}={I}_{re{l}_{OFF}}$. Furthermore, in such condition the fraction of dye molecules bound to lipids cannot increase and we assume that it does not decrease either, because the lipophilic tail of FM4-64 molecules maintains them bound to the lipid membrane. Consequently, in that case, ${\alpha }_{RO{I}_{ON}}={\alpha }_{RO{I}_{OFF}}$. From the last two equalities and Equations 23 and 24, it is straightforward to show that

25 $${{\rm{\Phi }}}_{{a}_{ON}}={{\rm{\Phi }}}_{a}$$

Which means that the NIR laser is not affecting the quantum yield of FM4-64 when bound to lipids. Using Equations 23, 24 and 25, the intensity ratio *Q*_*I*_ defined in Equation 2 becomes

26 $${Q}_{I}\equiv \frac{{I}_{re{l}_{ON}}}{{I}_{re{l}_{OFF}}}=\frac{{\alpha }_{RO{I}_{ON}}}{{\alpha }_{RO{I}_{OFF}}}$$

#### Analysis Software

Data were analysed using ImageJ (version 2.0.0) and a series of self-developed packages and programs written in Jython and in Python.

## Electronic supplementary material

Figure S1

## Data Availability

Data and analysis routines are available from the corresponding author upon reasonable request.
