# Supplementary material for: Effects of near infrared focused laser on the fluorescence of labelled cell membrane
Source: Sci Rep. 2018 Dec 5;8:17674. doi: 10.1038/s41598-018-36010-1 (PMC6281678; doi:10.1038/s41598-018-36010-1)
Supplement: Supplementary file 1 — Figure S1 [file 41598_2018_36010_MOESM1_ESM.pdf]

Effects of near infrared focused laser on the fluorescence of labelled cell membrane

Remy Avila,[1,2,*], Elisa Tamariz,[3] Norma Medina-Villalobos,[2,3] Jordi Andilla,[2] María Marsal,[2] Pablo Loza-Alvarez[2]

[1]Centro de Física Aplicada y Tecnología Avanzada, Universidad Nacional Autónoma de México (UNAM), Juriquilla, Querétaro, México
[2]ICFO-Institut de Ciencies Fotoniques, The Barcelona Institute of Science and Technology, 08860 Castelldefels (Barcelona), Spain
[3]Instituto de Ciencias de la Salud, Universidad Veracruzana, Xalapa, Veracruz, México

*Corresponding author: remy@fata.unam.mx

Supplementary Figure S1

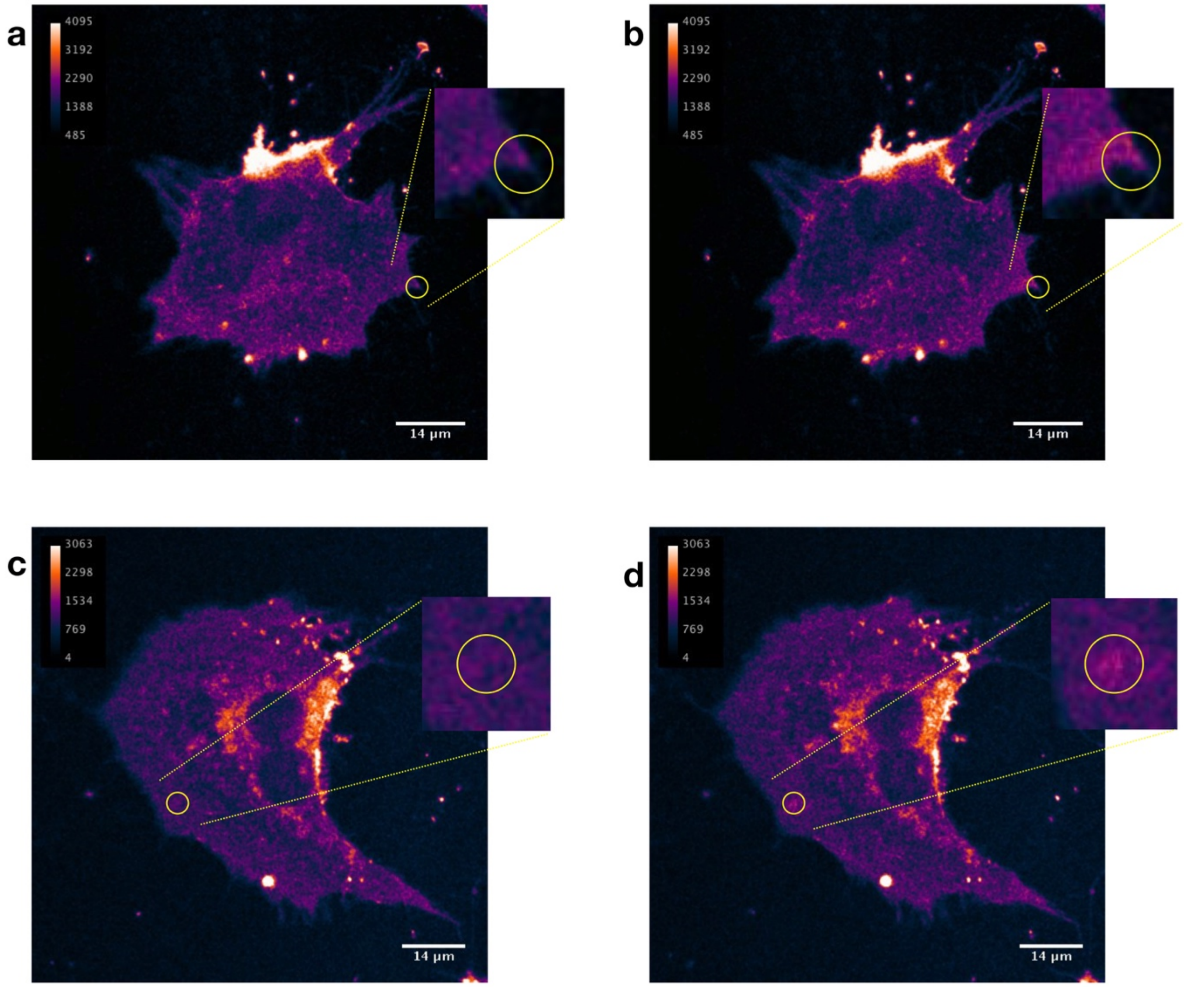

Figure S1: Examples of the effect of a 810-nm continuous wave focused laser on fluorescence intensity. **(a)** Confocal image of a 3T3 cell previously stained with FM 4-64, with the presence of dye molecules in the medium. NIR laser is turned off in this image. **(b)** Same as in (a) but with the 810-nm laser focused on the centre of the yellow circle which represents the ROI where intensity average is computed. An intensity rise is clearly seen by comparing images in the two magnified regions shown. **(c)** and **(d)** Same as (a) and (b), respectively, with another 3T3 cell.
